# The interpretation of change score of the pain disability index after vocational rehabilitation is baseline dependent

**DOI:** 10.1186/s12955-018-1000-1

**Published:** 2018-09-14

**Authors:** T. Beemster, C. van Bennekom, J. van Velzen, M. Reneman, M. Frings-Dresen

**Affiliations:** 1 Department of Rehabilitation Medicine, Center for Rehabilitation, University of Groningen, University Medical Center Groningen, Groningen, The Netherlands; 2 Department of Research and Development, Heliomare Rehabilitation Center, Wijk aan Zee, The Netherlands; 3 Amsterdam UMC, University of Amsterdam, Coronel Institute of Occupational Health, Amsterdam Public Health research institute, Amsterdam, The Netherlands

**Keywords:** Clinical relevance, Minimal important difference, Pain disability index, Occupational rehabilitation, Interpretation of change, Chronic pain

## Abstract

**Background:**

The Pain Disability Index (PDI) is a widely-used instrument to measure pain-related disability. The aim of this study was to assess the responsiveness and interpretation of change score of the PDI in patients with chronic musculoskeletal pain (CMP) at discharge of vocational rehabilitation.

**Methods:**

Retrospective data of patients with CMP who attended vocational rehabilitation between 2014 and 2017 was used. The anchor-based method was used to assess the responsiveness of the total sample and of PDI baseline quartile groups. A receiver operating characteristic curve was performed, including Area Under the Curve (AUC) and Minimal Important Change (MIC).

**Results:**

The PDI showed responsive to detect clinically relevant changes in pain-related disability at discharge of vocational rehabilitation (AUC 0.79). A PDI change score of 13 points (MIC 12.5) can be considered as a real change in pain-related disability for the total study sample, and a PDI change score of 7–20 points can be considered as a real change in pain-related disability for PDI lowest and highest baseline quartile scores.

**Conclusion:**

The PDI is responsive in patients with CMP at discharge of vocational rehabilitation. The interpretation of change score depends on PDI baseline score. Patients with a PDI baseline score of ≤27 should decrease minimal 7 points, patients with a baseline score between 28 and 42 should decrease minimal 15 points, and patients with a baseline score ≥ 43 should decrease minimal 20 points.

## Background

Chronic Musculoskeletal Pain (CMP) negatively affects quality of life, daily activities and social and working lives [1]. A decrease of pain-related disability is a desired outcome measure after rehabilitation for people with CMP [2]. A widely used and studied instrument to measure pain-related disability is the Pain Disability Index (PDI) [2, 3]. The PDI is a generic instrument: it can be administered to different patient groups, for example, chronic low back pain, fibromyalgia, cancer, or chronic widespread pain. The PDI is a valid [4–6] and reliable [6, 7] instrument. The utility of the PDI is high because it is easy to comprehend, it can be administered in a very short time, and it consists of only 7 questions [8].

However, the responsiveness, measurement error, and interpretability of change score of the PDI have scarcely been addressed. Responsiveness is the ability of a questionnaire to detect clinically important changes over time (for example, at discharge of a rehabilitation program) [9]. An outcome instrument should be able to distinguish clinically important change from measurement error [10]. The relation between responsiveness and measurement error should be made to interpret the (change) score of a questionnaire [10]. Nevertheless, to our knowledge, only one study [8] has assessed responsiveness and one other study [6] has assessed measurement error of the PDI. Good responsiveness (Area Under the Curve (AUC) of 0.76) was found in patients with chronic low back pain at discharge of a pain rehabilitation program in the Netherlands, and a minimal important change (MIC) of 8.5–9.5 points (depending on which anchor was used) was calculated [8]. In addition, a MIC value of 9.5 means that a decrease in PDI score of 9.5 points or more is a clinically meaningful improvement in pain-related disability. Measurement error, expressed in the Smallest Detectable Change (SDC), of 17.9 points was found in a sample with acute back pain, chronic low back pain, and widespread pain [6]. However, a connection between the MIC and the SDC (which refers to the interpretation of change score of the PDI), respectively, was not provided in the aforementioned studies. If we combine the MIC of 9.5 with the SDC of 17.9, we conclude that the PDI is responsive to change in patients with chronic back pain, but that it is uncertain if these are ‘real’ changes or are due to measurement error [11].

The aforementioned studies on responsiveness and measurement error were performed with patients attending pain rehabilitation in the Netherlands. It is unknown, however, what the responsiveness and interpretation of change score of the PDI is for patients at discharge of vocational rehabilitation (VR). Vocational rehabilitation is a “multi-professional evidence-based approach” that is provided in different settings, services, and activities to working age individuals with health-related impairments, limitations, or restrictions with work functioning, and whose primary aim is to “optimize work participation” [12]. However, it can be expected that the majority of patients referred to VR have paid work. In contrast, in pain rehabilitation samples, less than 50% of the patients have paid work [6, 13]. Since work is generally good for physical and mental health and well-being, and unemployment is associated with poorer physical and mental health and well-being [14], we expect that patients referred to VR are less disabled (i.e. lower PDI score) compared to patients referred to pain rehabilitation. We therefore assume that there is less room for improvement compared to patients with more severe pain-related disability and that this could result in lower MIC and change scores. This has, however, not yet been studied. Therefore, the aim of this study is to assess the responsiveness and interpretation of change score of the PDI in patients with chronic musculoskeletal pain at discharge of vocational rehabilitation.

## Methods

The COnsensus-based Standards for the selection of health Measurement INstruments (COSMIN) checklist was applied in the design of the study [9, 15, 16].

### Study sample

The study sample consisted of CMP patients who attended vocational rehabilitation (VR) between November 2014 and July 2017 in the Netherlands. Vocational rehabilitation is a multidisciplinary bio-psychosocial group-based program for workers with CMP and decreased work participation. The VR program is described in detail elsewhere [17]. The study sample was derived from seven vocational rehabilitation centers in the Netherlands. These seven centers are part of a nationwide network in the Netherlands and the outline and content of VR is similar at each center. The inclusion criteria for attending VR were: 1) being of working age (18 to 65 years); 2) suffering from subacute (6 to 12 weeks) or chronic (> 12 weeks) nonspecific musculoskeletal pain; 3) decreased work participation (i.e. part-time or full-time sick leave or reduced productivity while at work). The exclusion criteria were: 1) not motivated to participate in the multidisciplinary group-based program; 2) psychiatric disorders; 3) physical disorders with the expectation that tissue and function recovery will take place at normal rates; and 4) conflict situations with employer. Extra inclusion criteria for this study were: 1) being able to complete questionnaires in Dutch; and 2) having completed the Pain Disability Index at baseline and discharge of VR.

### Procedures

Data were collected using a core set of standardized web-based patient-reported questionnaires [18]. For this study, we only used the questionnaires on sample characteristics, including Pain Disability Index, assessed at baseline (T0) and discharge (T1); and Global Perceived Effect, assessed at T1 only. At T0 and T1, patients received an email with login data and the request to complete questionnaires (at home) on a website. Baseline questionnaires were sent to patients 1–2 weeks before a multidisciplinary screening, and the discharge questionnaires were sent to patients 1 week before discharge date. Because this study contains routinely collected and anonymous data of care as usual programs, the Medical Ethical Committee of the Academic Medical Center, Amsterdam, the Netherlands, authorized this study and decided that a full application was not required (reference number: A1 17.405).

### Outcome instrument: the pain disability index

The Pain Disability Index (PDI) is a 7-item questionnaire to investigate the magnitude of self-reported pain-related disability, independent from region of pain or pain-related diagnosis. The items of the questionnaire are assessed on a 0–10 numeric rating scale in which 0 means no disability and 10 is maximum disability. The sum of the seven items equals the total score of the PDI, which ranges from 0 to 70, with higher scores reflecting higher interference of pain with daily activities. The PDI measures family / home responsibilities, recreation, social activity, occupation, sexual behavior, self-care and life support activity [3]. Missing items were resolved as follows: patients were allowed to miss no more than 1 question on the PDI. In this case, the missing value was replaced by the patient cluster mean. As the PDI only consists of seven questions, the patient was excluded from the study [6] if the patient missed more than one question on the PDI.

### Anchor: Global perceived effect of treatment

A global perceived effect (GPE) item was used as the anchor (external criterion) in this study. An anchor is a global rating scale in which patients are asked, in a single question at follow-up, to indicate how much their pain has changed since baseline [19]. The pain anchor was assessed as follows: ‘How are your (pain) complaints at this moment compared to pre-treatment?’. The anchor was assessed on a 7-point Likert scale: extremely worsened, much worsened, little worsened, unchanged, little improved, much improved, completely improved.

### Data analyses

#### Responsiveness

Responsiveness in this study was defined as the ability of the PDI to detect clinically relevant changes in pain-related disability at discharge of vocational rehabilitation [9]. To calculate responsiveness we used the anchor-based receiver operating characteristics (ROC) method [20]. Sensitivity and specificity for change plotted by receiver operating characteristics (ROC) curve and Area Under the Curve (AUC) were calculated [10]. The AUC is the probability of correctly discriminating between improved and unchanged patients. When the AUC was more than 0.70, responsiveness was considered sufficient [10]. Minimal Important Change (MIC) was measured by determining the optimal cut-off point, i.e. the point where the sum of sensitivity and 1-specificity was maximal. Sensitivity and specificity range from 0 to 1.00, where higher numbers reflect higher sensitivity or specificity. Because the objective of the responsiveness analysis was to differentiate between improved and unchanged patients, the anchor scores were dichotomized into a subgroup with the score “improved” (much improved and completely improved) and a subgroup with the score “unchanged” (little worsened, unchanged and little improved) [8]. The group with the score “worsened” (much worsened and extremely worsened) was not included in the analyses (*n* = 14). We used the improved and unchanged groups to calculate the MIC [10, 20].

#### Baseline-dependent analyses

In a secondary analysis we stratified the analysis on PDI baseline quartile scores, to assess whether the level of pain-related disability on baseline had a modifying effect on the MIC. Based on earlier research [21, 22] we hypothesized that higher PDI scores at baseline (that is, more disabled patients thus higher PDI score) had more room for improvement, including higher change scores and MIC values compared to patients with lower baseline scores.

#### Floor and ceiling effects

Floor or ceiling effects were considered to be present if more than 15% of the respondents achieved the lowest or highest possible score (0–70, respectively) [10]. We gave a positive rating for (the absence of) floor and ceiling effects if no floor or ceiling effects were present in the PDI baseline quartiles [10].

#### Measurement error

Measurement error was analyzed by calculating the Standard Error of Measurement (SEM = SD√1-ICC) [23]. The SD was determined from an ANOVA analysis with the formula (√(SStotal /(n-1)) [10, 23]. As proposed by Terwee et al. [11], we derived the SD from our study sample for the patients with a non-significant change in PDI score (PDI total score T1 – PDI total score T0 = *p* > 0.05). Independent samples T test showed a non-significant change in PDI score when the PDI change score ranged from − 6 to + 6. The ICC of the SEM formula was obtained from a study with a similar study sample [6]. In a next step, the SEM was converted into the smallest detectable changes at individual level (SDCindividual = 1.96*√2*SEM). This number reflects the smallest within-person change in a score that can be considered to be a real change above any measurement error within one individual. In the final step, the SDC individual was converted into the smallest detectable change for a group (SDC group) by dividing SDC individual by √n.

#### Interpretability

**Fig. 1:**
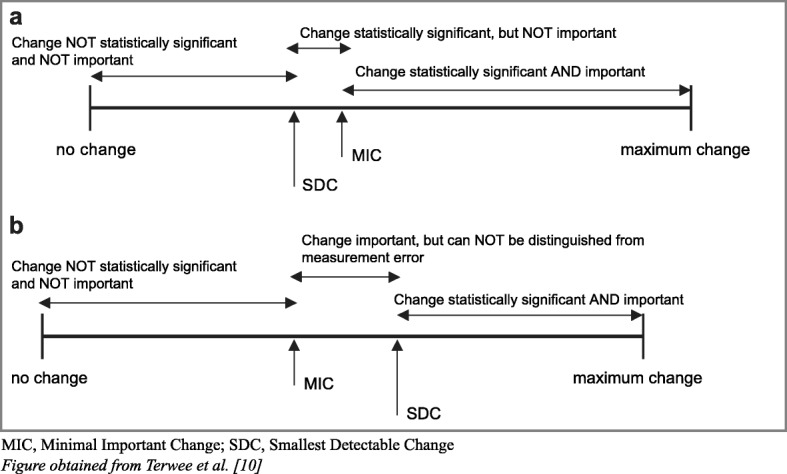
Interpretation of PDI change scores. MIC, Minimal Important Change; SDC, Smallest Detectable Change. Figure obtained from Terwee et al. [10]

Interpretability is defined as the degree to which one can assign qualitative meaning to quantitative scores [10]. To enhance interpretability, we will present baseline scores and change scores of various (sub)groups. For the interpretability of change scores, we calculated mean changes and 95% confidence intervals of mean changes of the total study sample and of the PDI baseline quartiles. We gave a positive rating for a real change in decrease of pain-related disability when the PDI change score was larger than the SDC, and if the SDC was smaller than the MIC [10, 19] (see Fig. 1).

All analyses were performed using SPSS 23 for Windows (SPSS Inc., Chicago, USA). The demographic data of the individuals were described by means and standard deviations (SD), or inter-quartile range in the case of no normal distribution. The assumption of normal data distribution was visually verified using histograms and QQ-plots.

## Results

A total of 341 patients completed the PDI questionnaire on baseline and discharge. Mean age was 46.5 (±10.9) years, and 57% of the patients were woman. Ninety-one percent of the patients were employed and 63% were on sick leave in the preceding month prior to baseline measurement. Patients suffered from 3.4 (±2.4) pain locations, which were located in the back (76%), lower extremities (35%) and upper extremities (29%). Seventy-four percent had pain complaints for longer than 6 months. The average pain score was 5.4 (±2.3), the worst pain score was 6.8 (±2.5) and the PDI mean score was 34.7 (±11.7). Mean duration between baseline questionnaires and the start of VR was 8 ± 4.4 weeks and mean duration between the start of VR and completion of the discharge questionnaires was 15 ± 1.1 weeks. Table 1 shows all background characteristics of the study sample.

**Table 1:** Characteristics of the study sample

	Unit of measurement	Vocational rehabilitation
		(*n* = 351)
Age (years)	Mean (sd)	46.5 (10.9)
Gender (female)	%	57.1
Education		
Low	%	15.1
Medium	%	54.0
High	%	24.9
Other	%	6.0
Work situation		
Employed	%	90.6
Student	%	0.6
Benefit	%	2.6
Other	%	6.3
Sick leave in the past month (yes)	%	63.4
Number of pain locations ^a-c^	Mean (sd)	3.4 (2.4)
	Median (IQR)	3 (1–5)
Pain location		
Spine (yes) ^a^	%	76.1
Lower extremities (yes) ^b^	%	35.0
Upper extremities (yes) ^c^	%	29.1
Pain duration		
1–3 months	%	7.4
3–6 months	%	18.9
0.5–1 year	%	23.4
1–2 year	%	19.1
2–5 year	%	14.9
More than 5 years	%	16.3
Pain average past week (0–10) ^d^	Mean (sd)	5.4 (2.3)
Pain worse past week (0–10) ^d^	Mean (sd)	6.8 (2.5)
PDI score (0–70) ^e^		
Total sample		
Baseline	Mean (sd)	34.7 (11.7)
	Range	3–60
Discharge	Mean (sd)	24.2 (14.1)
Mean change ^f^	Mean (sd)	−10.5 (13.8)^*^
	95% CI of mean change	9.1–12.0
Baseline PDI Q1		
Baseline	Mean (sd)	19.3 (6.2)
	Range	3–27
Discharge	Mean (sd)	16.4 (12.2)
Mean change	Mean (sd)	−2.9 (12.3)^*^
	95% CI of mean change	0.3–5.5
Baseline PDI Q2		
Baseline	Mean (sd)	32.0 (2.1)
	Range	28–35
Discharge	Mean (sd)	21.0 (11.8)
Mean change	Mean (sd)	−11.0 (11.7)^*^
	95% CI of mean change	8.4–13.5
Baseline PDI Q3		
Baseline	Mean (sd)	38.9 (2.1)
	Range	36–42
Discharge	Mean (sd)	28.0 (13.5)
Mean change	Mean (sd)	−10.9 (13.8)^*^
	95% CI of mean change	7.9–13.9
Baseline PDI Q4		
Baseline	Mean (sd)	48.8 (4.5)
	Range	43–60
Discharge	Mean (sd)	31.4 (13.6)
Mean change	Mean (sd)	−17.5 (13.4)^*^
	95% CI of mean change	14.6–20.3

*SD* standard deviation, *PDI* pain disability index, *IQR* interquartile range, *Q* quartile

^*^Significant change between baseline (T0) and discharge (T1) (*p* < 0.05)

^a^Spine, low back, upper back, neck and/or shoulder pain

^b^Lower extremities, hip(s), upper leg(s), and/or ankle(s)

^c^Upper extremities, arm(s), and/or hand(s) or finger(s)

^d^0 = no pain, 10 = worst possible pain

^e^0 = no disability, 70 = maximum disability

^f^PDI discharge score – PDI baseline score

### Responsiveness

**Fig. 2:**
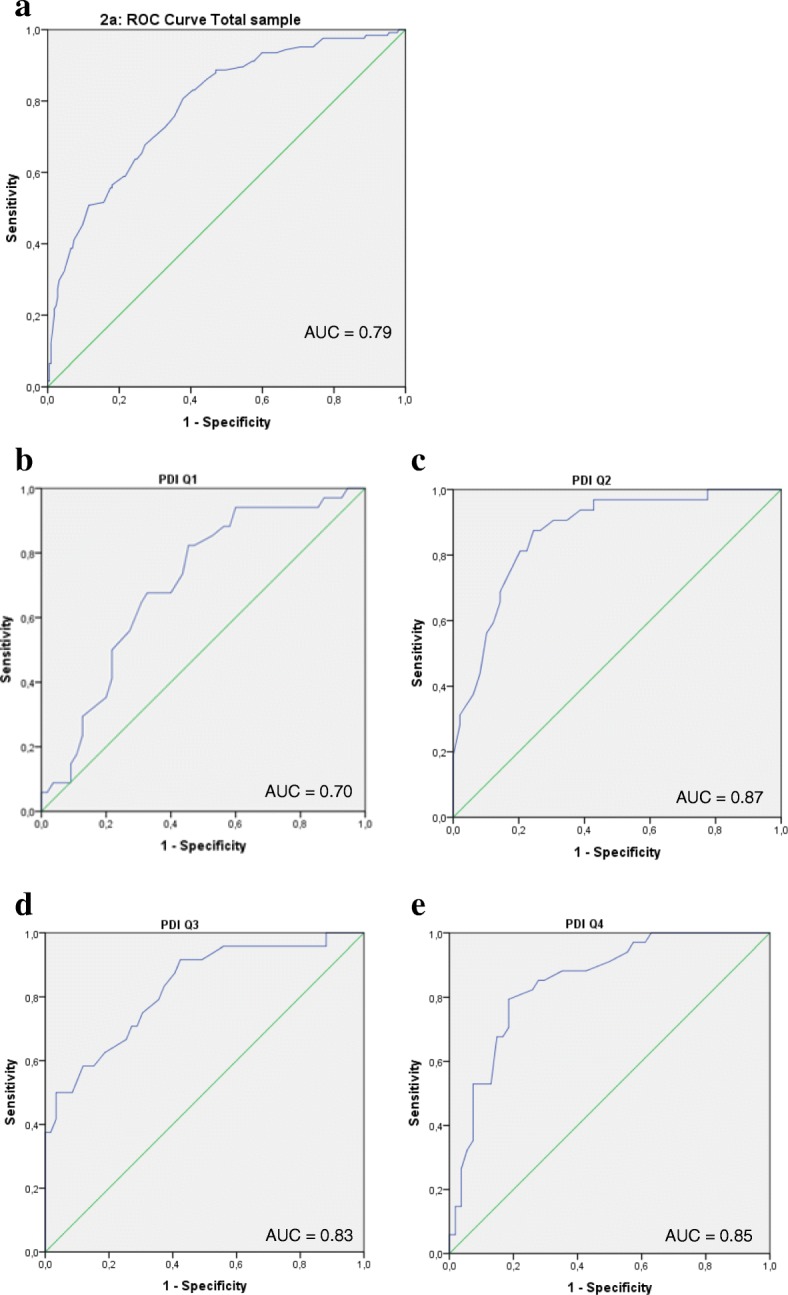
ROC curves of the PDI total sample and baseline quartiles. ROC, receiver operating characteristic; PDI, Pain Disability Index; Q, quartile; AUC, area under the curve. **a** ROC-curve of total study sample (*n* = 341). **b** ROC-curve of the sample with PDI baseline quartile 1 score (*n* = 89). **c** ROC-curve of the sample with PDI baseline quartile 12 score (*n* = 81). **d** ROC-curve of the sample with PDI baseline quartile 13 score (*n* = 83). **e** ROC-curve of the sample with PDI baseline quartile 14 score (*n* = 88)

The responsiveness parameters (AUC, MIC, sensitivity and specificity) of the total study sample and the baseline quartile scores are presented in Table 2, and the corresponding ROC curves are presented in Fig. 2. The AUC of the total sample was 0.79 (0.74–0.84), with a sensitivity of 0.68, a specificity of 0.73, and a corresponding MIC of 12.5 (Fig. 2a). The AUC of PDI baseline quartile 1 was 0.70 (0.59–0.81), with a sensitivity of 0.68, a specificity of 0.67, and a corresponding MIC of 6.5. The AUC of PDI baseline quartile 2 was 0.87 (0.79–0.95), with a sensitivity of 0.81, a specificity of 0.80, and a corresponding MIC of 14.5. The AUC of PDI baseline quartile 3 was 0.83 (0.73–0.93), with a sensitivity of 0.71, a specificity of 0.73, and a corresponding MIC of 14.5. The AUC of PDI baseline quartile 4 was 0.85 (0.77–0.93), with a sensitivity of 0.79, a specificity of 0.81, and a corresponding MIC of 19.5. In summary, the mean AUC of the total sample and of all PDI quartiles was sufficient, and only for quartile 1 the 95% confidence interval of the AUC felt bellow the cut off of 0.70, indicating slightly insufficient responsiveness for this quartile (also indicated by the shape of the ROC curve (Fig. 2b)).

**Table 2:** Responsiveness parameters PDI

Parameter	Total sample	Baseline PDI Q1 (3–27)	Baseline PDI Q2 (28–35)	Baseline PDI Q3 (36–42)	Baseline PDI Q4 (43–60)
Improved (N)	124	34	32	24	34
Stable (N)	217	55	49	59	54
AUC (CI)	0.79 (0.74–0.84)	0.70 (0.59–0.81)	0.87 (0.79–0.95)	0.83 (0.73–0.93)	0.85 (0.77–0.93)
MIC	12.5	6.5	14.5	14.5	19.5
Sensitivity	0.68	0.68	0.81	0.71	0.79
Specificity	0.73	0.67	0.80	0.73	0.81
SEM	1.2	1.2	1.2	1.2	1.2
SDC individual	3.4	3.4	3.4	3.4	3.4
SDC group	0.3	0.3	0.3	0.3	0.3

*PDI* pain disability index, *Q* quartile, *AUC* area under the curve, *CI* confidence interval, *MIC* minimal important change, *SEM* standard error of measurement, *SDC* smallest detectable change

### Floor and ceiling effects

Floor and ceiling effects were absent in this study. The PDI total baseline score (min-max) was 3–60; 2.6% of the study sample had a total PDI baseline score < 10 and 0.3% (1 person) of the study sample had a total PDI baseline score of 60.

### Measurement error

The SEM was 1.2, the SDC for group level was 0.3 and the SDC for individuals was 3.4 (Table 2).

### Interpretability

The SDC individual was smaller than the MIC in the total sample and in all PDI baseline quartile subgroups (Table 2). Of the total study sample, 70% improved at or above the SDC individual and 42% improved at or above the MIC (Table 3). Of the baseline quartile subgroups, 55–82% improved at or above the SDC individual and 40–46% improved at or above the MIC. Table 4 shows the PDI baseline score of various (sub) groups.

**Table 3:** Change scores in relation to MIC and SDC

	Total sample *N* = 341	Baseline PDI Q1 (3–27) *N* = 89	Baseline PDI Q2 (28–35) *N* = 81	Baseline PDI Q3 (36–42) *N* = 83	Baseline PDI Q4 (43–60) *N* = 88
Change ≥1 point (%)	76.5	65.2	79.0	74.7	87.5
MIC	−12.5	−6.5	−14.5	−14.5	−19.5
≥MIC (%)	41.9	46.1	44.4	39.8	42.0
SDC individual	3.4	3.4	3.4	3.4	3.4
≥SDC individual (%)	69.8	55.1	74.1	68.7	81.8
MIC > SDC	Yes	Yes	Yes	Yes	Yes

*PDI* pain disability index, *Q* quartile, *MIC* minimal important change, *SDC* smallest detectable change

**Table 4:** Reference values baseline PDI scores

Diagnosis	N	PDI score Mean (SD)	Source
Chronic musculoskeletal pain	351	34.7 (11.7)	Present study
General population	2510	6.8 (11.4)	Mewes 2009 [31]
Acute back pain	178	38.0 (15.9)	Soer 2013 [6]
Chronic back pain	242	34.6 (13.8)	Soer 2012 [8]
Chronic low back pain	425	36.5 (13.8)	Soer 2013 [6]
Chronic pain	4867	38.9 (13.3)	Köke 2017 [13]
Widespread pain	365	41.4 (10.9)	Soer 2013 [6]
Pain average past week (0–10)			
Patients with pain score 1–4	589	27.6 (13)	Köke 2017 [13]
Patients with pain score 5–6	1291	34.7 (11.5)	Köke 2017 [13]
Patients with pain score 7–10	2759	43.2 (12.2)	Köke 2017 [13]

*PDI* pain disability index, *SD* standard deviation

## Discussion

The results show that the PDI is responsive to detect clinically relevant changes in pain-related disability at discharge of vocational rehabilitation (AUC 0.79). A PDI change score of 13 points (MIC 12.5) can be considered as a real change in pain-related disability for the total study sample, and a PDI change score of 7–20 points can be considered as a real change in pain-related disability for PDI lowest and highest baseline quartile scores.

The responsiveness of the total study sample is in line with others [8] who found an AUC of 0.76 in patients with chronic back pain. However, the MIC of this study was 9.5 [8]. Because the sample size, external anchor’s (both 7-item Likert scale), and PDI version (both Dutch language versions) were similar amongst both studies, we hypothesize that the difference in MIC might be caused by the difference in mean change score, namely 10.5 in the current study and 6.8 in the other study [8]. This difference in mean change score might be affected by the different sample characteristics, settings, and interventions, applied in the other study; VR on the one hand versus multidisciplinary rehabilitation, surgery, or anesthesiology [8]. Another explanation for the difference in MIC might be caused by the different ways in questioning the GPE anchor item, which was formulated in the current study as follows: “How are your (pain) complaints at this moment compared to pre-treatment?”, and which was formulated in the other study as follows: “How much did your treated complaints change compared with pretreatment level?”. Finally, the same data was collected in the present study between 2014 to 2017; despite the passage of time, the diversity of centers and professionals involved in the collection of data. These factors also could have influenced the findings on responsiveness. In summary, the different MIC and change scores between the present and discussed study show that the MIC and change score can differ per sample and setting.

The mean change score of the present study (10.5) is somewhat higher compared to a study that found a mean change score in PDI of 9.4 in patients with chronic pain after a multidisciplinary pain program [19]. This is surprising, because the study mentioned had a higher PDI baseline value, namely 37.8, which implicates more room for change, which we actually showed in the present study. Another study showed a mean change score in PDI of 14.0 (baseline score 47.6) in workers’ compensation claimants with musculoskeletal disorders after a functional restoration program [24]. This PDI change score is slightly lower compared with the mean change score of 17.5 of the fourth quartile of the present study, but it supports our finding that interpretation of the PDI change score is baseline dependent.

The interpretation of change score of the PDI can be interpreted as a “real” change in pain-related disability if the mean change score is at or above the MIC and if the SDC for individuals does not exceed the MIC (Fig. 1, Table 2). It is difficult to compare our results with other studies, however, for two reasons. Firstly, we are only aware of one study that found an SDC of 17.9 in patients with acute back pain, chronic low back pain, and widespread pain [6]. The huge discrepancy compared with the current study (SDC 3.4) can be explained by the fact the study in question used the standard deviation of the mean PDI baseline score in the calculation of the SDC (personal communication with first author (RS)). We suppose that it is important for the calculation of the SDC to take the variability between time points into account [11]. Secondly, change scores of longitudinal cohort studies are regularly reported on group level (i.e. mean scores), whereas it is much more interesting to report the percentage of improved patients (according to the MIC), because this “… provides readers with values which are more easily understood and additional information to help them decide whether a treatment should be used.” [22].

The baseline PDI score of the current study is similar compared to patients with chronic back pain [6, 8], but somewhat lower compared to patients with chronic pain and widespread pain. One reason for this difference might be a difference in patients executing paid work, which was 91% in the current study and 48 and 43% in chronic pain and widespread pain [6, 13]. Another difference might be due to a difference in pain baseline score of the present study compared with the chronic and widespread pain samples (5.4 versus 6.7 and 6.9, respectively). Köke et al. showed that higher pain score on baseline is related to significantly higher PDI baseline scores [13].

### Methodological considerations

The first methodological consideration of this study was the assessment of the MIC. Two common methods can be used to calculate the MIC: the distribution-based method and the anchor-based method [20]. In the distribution-based method, 50% of the standard deviation of the baseline score (0.5*SD) of the measurement instrument serves as the MIC. In the anchor-based method an external anchor is used as the “gold standard” to discriminate between improved and unchanged persons, and the MIC can be obtained with an ROC curve. Because the MIC can be derived from the sensitivity and specificity provided with an ROC curve, the MIC can be used in scientific research and clinical practice as a cutoff point to determine the number of patients that have significantly changed. Patients with a change score greater than or equal to MIC can be called “responders”. With this method, the difference in percentages of responders between treatment groups can be determined [11]. Because of the aforementioned advantage, and because this method is recommended [20, 25–28], we used the anchor-based method in the present study. The second methodological consideration was how we dichotomized the anchor item into changed and unchanged groups, which we used for the calculation of the MICs. In the present study, the changed group consisted of patients who were “much improved” and “completely improved” and the unchanged group consisted of patients who were “little worsened”, “unchanged”, and “little improved”. Other papers, however, state that only a “little improved” group can serve as the (minimal important) change group [28, 29], or “little improved”, “much improved” and “completely improved” as the changed group [20]. We, however, agree with Ostelo et al. who stated that “…“little improvement” is in the range of natural fluctuation, and that an “important” improvement should be greater than these (unimportant) fluctuations” [30]. However, it is important to notice that the type of anchor-dichotomization directly influences the AUC and MIC. Therefore, the results of the present study must be interpreted with caution because the used cutoff has a high influence on the findings [20, 30]. The third and final methodological consideration was the number of baseline (sub)groups. We decided a priori to apply four subgroups (i.e. quartiles), because we had enough power. The number of four subgroups used in the present study was arbitrary, however. Nevertheless, there are no guidelines for conducting a particular number of (sub)groups based on baseline score, and there are as yet no subgroup scores known for the PDI based on pain-related disability (for example “low”, “intermediate” and “high” pain-related disability subgroups). Since the second and third baseline quartile of the present study showed similar MICs and mean change scores, future studies might propose to assess the responsiveness of three PDI baseline subgroups based on interquartile range (25th, 50th, and 75th percentile).

### Clinical message

Practitioners can use the following cutoff scores to decide if a PDI change score is clinically relevant at discharge of VR: patients with a baseline score of ≤27 should decrease minimal 7 points, patients with a baseline score between 28 and 42 should decrease minimal 15 points, and patients with a baseline score ≥ 43 should decrease minimal 20 points.

## Conclusion

The PDI is a responsive questionnaire which can detect real change in decrease of pain-related disability in patients with CMP at discharge of vocational rehabilitation. Future research should focus on assessing the SDC and the MIC of the PDI in various patient samples and settings. Also, when using longitudinal cohorts, researchers are encouraged to report the portion of the sample with a change score at or above the MIC since this will enhance comparability and clinical relevance.

## Abbreviations

AUC: Area under the curve
CMP: Chronic musculoskeletal pain
COSMIN: COnsensus-based Standards for the selection of health Measurement INstruments
GPE: Global perceived effect
ICC: Intraclass correlation coefficient
MIC: Minimal Important Change
PDI: Pain Disability Index
QQ plot: Quantile-quantile plot
ROC: Receiver operating characteristics curve
SD: Standard deviation
SDC: Smallest Detectable Change
SEM: Standard Error of Measurement
SPSS: Statistical Package for the Social Sciences
SStotal: Sum of squares total
VR: Vocational rehabilitation
